# Focal Adhesion Kinase Inhibitor Inhibits the Oxidative Damage Induced by Central Venous Catheter via Abolishing Focal Adhesion Kinase-Protein Kinase B Pathway Activation

**DOI:** 10.1155/2021/6685493

**Published:** 2021-03-01

**Authors:** Yanru Wang, Sulan Lin, Ping Jiang, Yunlin Song, Yanjie Zhao, Yujian Zheng

**Affiliations:** ^1^Department of Occupational and Environmental Health, Public Health School, Xinjiang Medical University, Urumqi 830000, China; ^2^Department of Basic Nursing, Nursing School, Xinjiang Medical University, Urumqi 830000, China; ^3^Department of Pathophysiology, School of Basic Medicine, Xinjiang Medical University, Urumqi 830000, China; ^4^Department of Critical Care Medicine, The First Affiliated Hospital, Xinjiang Medical University, Urumqi 830000, China

## Abstract

The vascular injury induced by central venous catheter (CVC) indwelling is the basis for the occurrence and development of CVC-related complications, such as phlebitis, venous thrombosis, and catheter-related infections. Focal adhesion kinase (FAK) and FAK-protein kinase B (AKT) signaling pathway are of great significance in tissue repair after trauma. Here, we investigated the role and mechanism of the FAK inhibitor (1,2,4,5-phenyltetramine tetrahydrochloride (Y15)) in oxidative damage caused by CVC. EA.hy926 cells were divided into the control group (normal control), CVCs+scratches group (the intercepted CVC segments coculturing with scratched EA.hy926 cells), and CVCs+scratches+Y15 group (Y15 was added to the cell culture supernatant with CVCs + scratches at a final concentration of 50 *μ*mol·L^−1^). New Zealand rabbits were randomly divided into the control group (normal control), CVC group (CVC was inserted through the rabbit's right jugular vein to the junction of the right atrium and superior vena cava), and CVC+Y15 group (CVC was immersed in a 50 *μ*mol·L^−1^ Y15 solutions before insertion). The levels of markers and proteins related to oxidative damage in cells, cell culture supernatant, serum, and external jugular vein were measured by commercial kits and western blot, respectively. We found that Y15 treatment significantly decreased ROS and MDA levels and increased cell viability, NO, and SOD levels in a time-dependent manner in rabbit serum and cell culture supernatant. In addition, Y15 effectively reduced the CVC-induced pathological changes of damaged vascular tissues. Y15 also downregulated the levels of p-FAK Tyr 397 and p-Akt Ser 473 in damaged external jugular vein and EA.hy926 cells. These findings suggest that Y15 alleviated CVC-induced oxidative damage to blood vessels by suppressing focal FAK-Akt pathway activation.

## 1. Introduction

A common operation in the intensive care unit is to insert and retain the central venous catheter (CVC). The catheters are usually used for intravenous administration of blood products, hypertonic solutions, liquid nutrition, and other therapies to critically ill patients. However, CVC-induced complications (including infection and venous thrombosis) are important reasons for prolonging the patient's hospital stay. Moreover, these complications not only increase the economic burden of patients but also cause a high risk of death [1]. Although clinical measures have been taken, including the use of antibiotic flushing or sealing, the use of antibacterial catheters and, drug-eluting catheters, to prevent or reduce related complications, these complications still persist [2–4].

The oxidative stress caused by CVC is attributed to the occurrence and development of CVC-related complications, such as phlebitis [5, 6], venous thrombosis [7], and infection [8]. Himmelfarb et al. reported that CVC implantation was an important prooxidant factor leading to catheter-related complications and the development of cardiovascular diseases [6]. Chen et al. found that placing CVC in the right external jugular vein of rats caused endothelial cell damage, collagen exposure, and oxidative stress due to the upregulation of microRNA-92a, which further led to thrombosis [9]. In addition, the use of CVC reduces the levels of antioxidant enzymes and paraoxonase in patients, thereby promoting oxidation and inflammation and enhancing the development of CVC-related infections [10]. However, the pathways and underlying mechanisms for reducing the oxidative damage caused by CVC are still unclear.

The imbalance in the production and consumption of reactive oxygen species (ROS) leads to oxidative stress in cells. Oxidative stress biomarkers (including ROS, nitric oxide (NO), malondialdehyde (MDA), and superoxide dismutase (SOD)) can reflect the degree of cell oxidative damage [11–13]. Therefore, it is necessary to adopt effective antioxidant strategies to reduce the production of ROS, thereby preventing oxidative damage caused by CVC.

In recent years, it has been reported that the focal adhesion kinase (FAK) signaling pathway plays a regulatory role in cancer [14], posttraumatic repair [15], oxidative stress, and inflammatory response [16]. Additionally, ROS and cytokines that cause endothelial dysfunction can induce FAK family activation [17, 18]. Thus, the inhibition of both phosphorylation of FAK and protein kinase B (AKT) should play an important role in antioxidation, anti-inflammation, and tumor microenvironment improvement [19]. Inhibition of Akt (Thr308 and Ser473) phosphorylation through the phosphatidylinositol 3-kinase (PI3K)/Akt pathway significantly reduces acute lung injury [20]. Thus, we hypothesize that inhibiting activation of FAK and Akt could alleviate the oxidative damage.

Y15 (1,2,4,5-benzenetetramine tetrahydrochloride) is a novel small molecule FAK phosphorylation inhibitor that targets the Y397 of FAK. It decreases phosphorylation of Y397-FAK and total phosphorylation of FAK [21]. Nonetheless, the effects of Y15 on oxidative injury induced by CVC remain unknown. Here, in this study, we established two models of oxidative damage induced by CVC or CVCs + scratches, including oxidative damage of external jugular vein or EA.hy926 cells. The role of Y15 on oxidative damage caused by CVC was evaluated. The underlying mechanisms involving the FAK-Akt signaling pathway were also analyzed and discussed.

## 2. Materials and Methods

### 2.1. Reagents

CVCs were purchased from Yixinda Medical New Technology Co., Ltd. (Shenzhen, China). Y15 were purchased from MedChem Express (Monmouth Junction, NJ, USA). Fetal bovine serum (FBS), Dulbecco's Modified Eagle's Medium (DMEM), and dimethyl sulfoxide (DMSO) were from Hyclone (Austria). Penicillin and streptomycin were from Sigma-Aldrich (St. Louis, MO, USA). 3-(4,5-Dimethylthiazol-2-y1)-2,5-diphenyltetrazolium bromide (MTT) was purchased from Solarbio (Beijing, China). Detection kits for ROS, MDA, NO, and SOD were all from Nanjing Jiancheng Bioengineering Institute (Nanjing, China). Mouse anti-rabbit antibodies included FAK (Cat# abx133010, Abbexa Ltd.), phospho-FAK (Tyr397) (Cat# FM1211, ECM Biosciences), Akt (Cat# orb11276, Biorbyt), and phospho-Akt (Ser473) (Cat# 05-1003, Merck Millipore). Rabbit anti-human antibodies for FAK (Cat# 3285), phospho-FAK (Tyr397) (Cat# 8556), Akt (Cat# 9272), and phospho-Akt (Ser473) (Cat# 4058) were all from Cell Signaling Technology. Secondary antibodies (anti-mouse IgG [Cat# PV-6002], anti-rabbit IgG [Cat# ZB-2301], mouse anti-GAPDH [Cat# AB-M-M001], and rabbit anti-GAPDH [Cat# AB-P-R001]) were all from Goodhere Biotechnology Co., Ltd.

### 2.2. Animals

Forty-five male New Zealand white rabbits with an average body weight of 2.5 ± 0.3 kg were provided by the Medical Animal Experimental Center at Xinjiang Medical University under production license number SYXK (New) 2014-0001. They were kept in standard conditions. All animal experimental procedures were approved by the Ethics Committee of Xinjiang Medical University.

### 2.3. Animal Treatments and Grouping

The rabbits were randomly divided into three groups, including the control group (*n* = 15), CVC group (*n* = 15), and CVC+Y15 group (*n* = 15; five rabbits each at weeks 2, 4, and 6 after CVC implantation). In the CVC and CVC+Y15 groups, the rabbits were anesthetized with 20% urethane at a dose of 5 mL/kg [22, 23]. A CVC/Y15-impregnated CVC (the CVC was immersed in Y15 solution (50 *μ*mol/L) for 5 min) was inserted into the right superior vena cava via the right external jugular vein to a depth of approximately 7 cm. After catheterization, the blood was drawn back via the CVC daily, and the tubes were flushed with 20 mL of 0.9% saline and 1% heparin sodium to seal the tubes. There was no intervention in the control group.

### 2.4. Sampling

After fasting for 12 h, rabbits were anesthetized by intravenous injection of 20% urethane (5 mL/kg, 10-20 mL/5-10 min) through the ear margin. Then, 20 mL of blood sample was collected from the common carotid artery. After centrifugation for 20 min (3000 rpm), the serum was collected. After the blood collection, overdose urethane was injected intravenously to euthanize the rabbit. The right external jugular veins were collected within 30 min after the rabbits were euthanized.

### 2.5. HE Staining

The right external jugular vein tissues were dissected and immediately fixed in 10% formaldehyde. HE staining was performed according to previous description [24]. Briefly, the vein tissues were washed with running water for 24 h and then embedded with paraffin. The embedded block was cut into pathological sections of approximately 5 *μ*m thickness. After deparaffinization, the sections were stained with hematoxylin for 15 min. After that, the sections were washed with water, restained with eosin solution for 5 min, dehydrated with alcohol, hyalinized, and mounted. Finally, the pathological changes in the vascular endothelium were observed under a light microscope.

### 2.6. EA.hy926 Cell Culture

The human umbilical vein cell line EA.hy926 was purchased from the Cell Bank of the Type Culture Collection of the Chinese Academy of Sciences (Shanghai, China). The cells were then cultured in DMEM medium supplemented with 10% FBS, 2 mmol/L of L-glutamine, 100 units/mL of penicillin, and 100 *μ*g/mL of streptomycin [25]. The cells were cultured at 37°C and 5% CO_2_.

### 2.7. Cell Treatment

EA.hy926 cells were divided into three groups. The control group was treated with 2 mL DMEM containing 10% FBS. The CVCs+scratches group was treated with 2 mL DMEM containing 10% FBS + 3 scratches + 3 segments of CVCs (1 cm). The CVCs+scratches+Y15 group was treated with 1.950 mL DMEM containing 10% FBS + 3 scratches + 3 segments of CVCs (1 cm) + 2 mmol/L of Y15 (50 *μ*L). For the scratches, the monolayer cells at the bottom of the well were scratched with a sterile ruler and a 10 *μ*L pipette tip after DMEM was added to each well. The final concentration of Y15 was 50 *μ*mol/L [26]. After cell treatment, cell culture supernatant was collected at 24, 48, and 72 h, respectively, to detect oxidative stress level.

### 2.8. MTT Assay

EA.hy926 cells were plated in 96-well plates at about 1 × 10^5^ cells per well and treated as above described for 24, 48, and 72 h. Then, 20 *μ*L of MTT (thiazolyl blue) was added into each well with a final concentration of 0.5 mg/m and incubated for 4 h at 37°C. After that, 150 *μ*L DMSO was added to solubilize the blue precipitate. The optical density of the cells in each group was then measured using a microplate reader (Thermo Fisher Scientific, Inc., Waltham, MA, USA) at OD 490 nm.

### 2.9. Tissue and Cell Protein Extraction

The right external jugular veins were washed, cut into pieces, and homogenized with radio-immunoprecipitation assay (RIPA) lysate (a 1% protease inhibitor cocktail) for 30 min. Then, the lysate was centrifuged twice at 4°C at 12,000 rpm for 12 min each time. The supernatant was collected. EA.hy926 cells were treated as previously described and then were subjected to cell lysis with RIPA buffer (containing protease and phosphatase inhibitors) on ice for 45 min. After centrifugation at 4°C for 20 min, the supernatant was collected.

### 2.10. Determination of ROS, SOD, NO, and MDA

The levels of ROS, SOD, NO, and MDA in rabbit serum and cell culture supernatant were quantified according to the manufacturer's instructions. Briefly, 50 *μ*L of standards or samples was added into each well. Then, 100 *μ*L of enzyme conjugate was added and incubated for 60 min at 37°C. After rinsing with PBST for 4 times, the development reagents were added for color development in the dark for 15 min at 37°C. After adding 50 *μ*L stop solution, the samples were read at OD450 nm using a microtiter plate reader within 15 min.

### 2.11. Western Blot

Total proteins of tissues and cells were separated with 12% SDS-PAGE and then transferred onto PVDF membranes. The PVDF membranes were then incubated with 5% nonfat dry milk for 1 h at room temperature. After washing, the membranes were then incubated with primary antibodies at 4°C for 24 h, including anti-FAK (1 : 1000), anti-phospho-FAK (Tyr397) (1 : 1000), anti-Akt (1 : 500), anti-phospho-Akt (Ser473) (1 : 2000), and anti-GAPDH (1 : 1000). After washing again, the membranes were incubated with secondary antibody of horseradish-peroxidase conjugated IgG (1 : 5000) for 2 h at room temperature. Finally, an enhanced chemiluminescent reagent was added to the membranes for color development. An autoradiography system (Bio-Rad, Germany) was used to observe the blots. The ImageJ software (V1.8.0; NIH, MD, USA) was used to analyze the gray vale of each protein [27]. The relative expression level of target protein was calculated as the ratio of the gray value of the target protein to that of GAPDH.

### 2.12. Statistical Analysis

Data was analyzed using the GraphPad Prism statistical software version 6.0 (GraphPad Software, Inc., USA). Data was expressed as the means ± standard deviation (SD). One-way/two-way ANOVA followed by the Bonferroni post hoc test was used to analyze differences among groups. A *p* value < 0.05 indicated a significant difference.

## 3. Results

### 3.1. Y15 Improves Pathological Changes of External Jugular Vein in Rabbits

**Figure 1:**
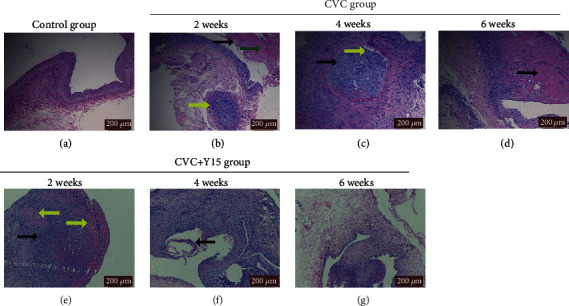
Histopathological changes of the right external jugular vein. HE staining was performed to evaluate the histopathological changes. Representative images are shown. (a) Control group (×10). (b) CVC group at week 2 (×10): vascular intima shedding (black arrow), subintimal hemorrhage (green arrow) and mucoid degeneration (yellow arrow). (c) CVC group at week 4 (×10): granulation tissue organization in venous lumen (black arrow) and inflammatory cell infiltration (yellow arrow). (d) CVC group at week 6 (×20): scar tissue formation under the intima (black arrow). (e) CVC+Y15 group at week 2 (×20: inflammatory cell infiltration (black arrow) and hyaline degeneration (yellow arrow). (f) CVC+Y15 group (×10) at week 4: vascular intima shedding (black arrow). (g) CVC+Y15 group at week 6 (×10). Scale bar: 200 *μ*m.

HE staining showed that the intima of the right external jugular vein in the control group was smooth and there was no obvious tissue damage (Figure 1(a)). However, the longer CVC retention time, the more severe the vascular injuries and inflammatory responses in the CVC group (Figures 1(b)–1(d)). After four weeks of intervention, the granulation tissue in venous lumen was organized, and there was the large number of inflammatory cell infiltration (Figure 1(c)). Six weeks later, the scar tissue in the vein was formed (Figure 1(d)). Y15 treatment markedly reduced inflammatory cell infiltration (Figure 1(e)). Although there was vascular intima shedding (Figure 1(f)), no thrombosis was observed (Figure 1(g)). The results showed that Y15 alleviated the pathological changes of external jugular vein induced by CVC.

### 3.2. Y15 Changes the Levels of ROS, MDA, NO, and SOD in Rabbits with CVC

**Figure 2:**
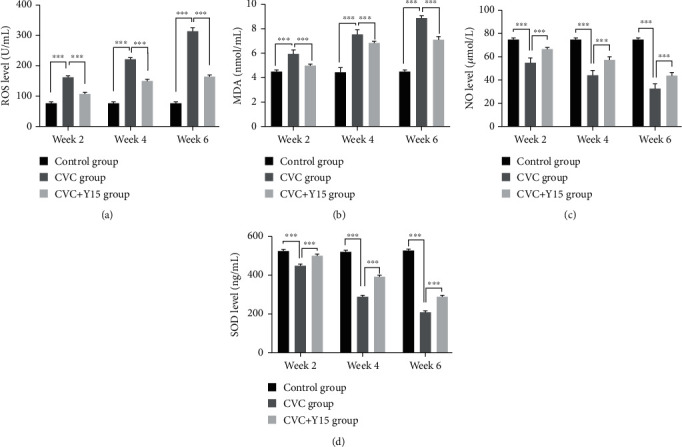
Y15-impregnated CVCs decreased the degree of oxidative damage of external jugular veins. ROS (a), MDA (b), NO (c), and SOD (d) levels in vein tissue were determined using ROS, MDA, NO, and SOD test kits, respectively. Data are presented as the mean ± SD (*n* = 5 for each time point per group). *p* values (*p* < 0.05) were calculated by two-way ANOVA analysis with the Bonferroni multiple comparison test. ^∗∗∗^*p* < 0.001.

The levels of ROS and MDA in the CVC group and CVC+Y15 group increased with the increase of CVC retention time. Statistically, at weeks 2, 4, and 6, the ROS and MDA levels of the CVC group were significantly higher than those of the control group (*p* < 0.001, Figures 2(a) and 2(b)). However, the CVC+Y15 group had significantly lower ROS and MDA levels at the same time point than the CVC group (*p* < 0.001, Figures 2(a) and 2(b)). In contrast, the levels of NO and SOD in the CVC group and the CVC+Y15 group decreased with the increase of the CVC retention time. The levels of NO and SOD were significantly lower than those of the control group (*p* < 0.001, Figures 2(c) and 2(d)). However, compared with the CVC group, NO and SOD levels in the CVC+Y15 group markedly increased (*p* < 0.001, Figures 2(c) and 2(d)). The results indicated that Y15 decreased ROS and MDA whereas it increased NO and SOD in the damaged external jugular vein.

### 3.3. Y15 Downregulates the p-FAK Tyr 397 and p-Akt Ser 473 Levels in Rabbits with CVC

**Figure 3:**
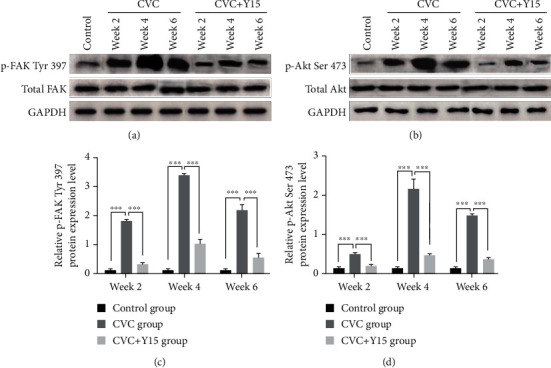
Western blot was performed to determine expression of total FAK, Akt, p-FAK Tyr 397, and p-Akt Ser 473. (a, b) The expression of total FAK, p-FAK Tyr 397, total Akt, and p-Akt Ser 473 in the CVC group and CVC+Y15 group. (c, d) The expression levels of p-FAK Tyr 397 and p-Akt Ser 473 at 2-week, 4-week, and 6-week time points were compared. Data are presented as the mean ± SD (*n* = 5 for each time point per group). Statistical significance was set at *p* < 0.05 and was determined by two-way ANOVA analysis was performed using Bonferroni multiple comparison test. ^∗∗∗^*p* < 0.001.

Western blot was conducted to detect protein levels. Relative p-FAK Tyr 397 and p-Akt Ser 473 levels at week 2, week 4, and week 6 in both the CVC group and CVC+Y15 group (p-FAK were observably higher than those of the control group (Figures 3(a)–3(d), *p* < 0.001). Moreover, the levels of p-FAK Tyr 397 and p-Akt Ser 473 in the CVC+Y15 group were significantly lower than those in the CVC group at each time point (*p* < 0.001). The results showed that Y15 downregulated the p-FAK Tyr 397 and p-Akt Ser 473 levels induced by damaged external jugular vein.

### 3.4. Y15 Improves the Viability of Damaged EA.hy926 Cells

**Figure 4:**
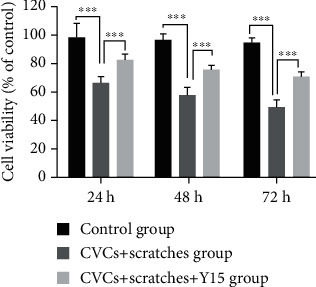
Effect of Y15 on the cell viability of damaged EA.hy926 cells. Cell viability was detected with MTT assay. Data are presented as the mean ± SD (*n* = 5 for each time point per group). Two-way ANOVA analysis was performed using the Bonferroni multiple comparison test to determine *p* values (*p* < 0.05). ^∗∗∗^*p* < 0.001.

Cell viability of EA.hy926 was analyzed with MTT assay. With the extension of the CVC retention time, the cell viability of the CVCs+scratches group and CVCs+scratches+Y15 group decreased. However, the cell viability of the CVCs+scratches group was significantly lower than that of the control group (*p* < 0.001, Figure 4). The cell viability at each time point in the CVCs+scratches+Y15 group was significantly higher than that in the CVCs+scratches group (*p* < 0.001, Figure 4). Thus, the results showed that the degree of cell damage in the CVCs+scratches+Y15 group was lower than that of the CVCs+scratches group.

### 3.5. Y15 Changes the Levels of ROS, MDA, NO, and SOD in Damaged EA.hy926 Cells

**Figure 5:**
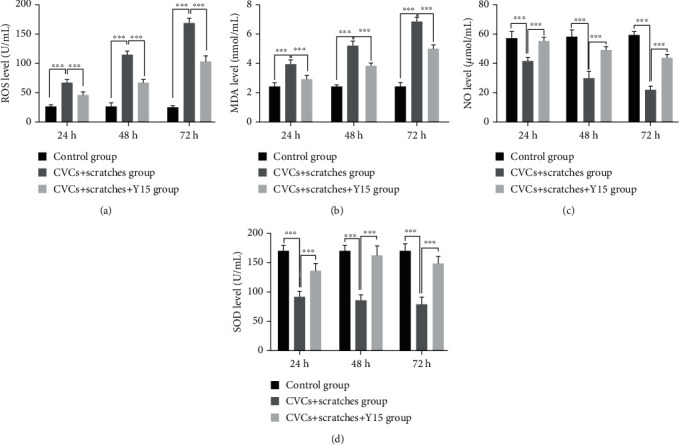
Treatment of damaged EA. hy926 cells with Y15 decreased ROS and MDA levels and increased concentration of SOD and NO. ROS (a), MDA (b), NO (c), and SOD (d) levels in cells were measured with ROS, MDA, SOD, and NO test kits. Data are presented as the mean ± SD (*n* = 5 for each time point per group). *p* values (*p* < 0.05) were calculated by two-way ANOVA analysis with the Bonferroni multiple comparison test. (a, b) ^∗∗∗^*p* < 0.001.

Generally, the ROS and MDA levels of cells in the CVCs+scratches group increased while those of the CVCs+scratches+Y15 group decreased. As shown in Figures 5(a) and 5(b), compared with the control group, the levels of ROS and MDA at each time point (24 h, 48 h, and 72 h) in the CVCs+scratches group were remarkably higher (*p* < 0.001). Compared with those of the CVCs+scratches group, those levels in the CVCs+scratches+Y15 group were significantly lower (*p* < 0.001). However, NO and SOD levels in the CVCs+scratches group were markedly lower than those of the control group at each time point (*p* < 0.001, Figure 5(c)). NO and SOD levels were significantly higher at each time point in the CVCs+scratches+Y15 group than those in the CVCs+scratches group (*p* < 0.001, Figures 5(c) and 5(d)).

### 3.6. Y15 Reduces the p-FAK Tyr 397 and p-Akt Ser 473 Levels in Damaged EA.hy926 Cells

**Figure 6:**
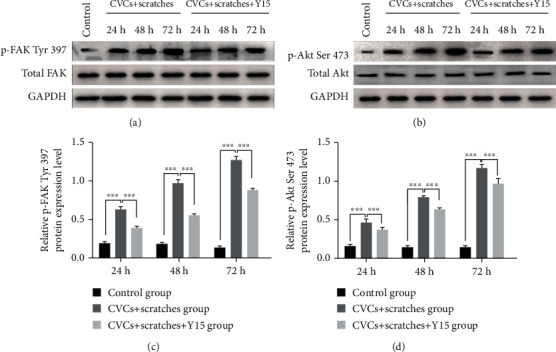
Y15 decreased protein levels of p-FAK, Tyr 397, and p-Akt Ser 473. Western blot was performed to determine expression of total FAK, Akt, p-FAK Tyr 397, and p-Akt Ser 473 at 24 h, 48 h, and 72 h. (a, c) The expression of total FAK and p-FAK Tyr 397 in the CVCs+scratches group and CVCs+scratches+Y15 group. (b, d) The protein levels of total Akt and p-Akt Ser 473. ^∗∗∗^*p* < 0.001.

As detected by Western blot, relative p-FAK Tyr 397 and p-Akt Ser 473 levels in both the CVCs+scratches group and the CVCs+scratches+Y15 group were significantly higher than those of the control group (*p* < 0.001, Figures 6(a)–6(d)) at the same time point. Moreover, the levels of p-FAK Tyr 397 and p-Akt Ser 473 at 24 h, 48 h, and 72 h in the CVCs+scratches+Y15 group were dramatically lower than those in the CVCs+scratches group at the same time points (*p* < 0.001 in p-FAK Tyr 397 levels and *p* < 0.01 in p-Akt Ser 473 levels between the two groups). These results suggested that the Y15 in the CVCs+scratches+Y15 group decreased the activation levels of FAK Tyr 397 and Akt Ser 473.

## 4. Discussion

FAK is reported to be involved in the occurrence and development of oxidative stress and inflammation [16]. When noxious stimuli act on cells, phosphorylated FAK or part of FAK-related signaling pathways can upregulate intracellular ROS levels, leading to abnormal cellular oxidative metabolism [28, 29]. Y15, an FAK inhibitor, can target the Y397 site of FAK and inhibits its autophosphorylation [21], thereby regulating NO, ROS, MDA, and SOD levels [30, 31] via inhibiting of Src/FAK, PI3K/Akt/endothelial nitric oxide synthase (eNOS), and FAK/Akt/eNOS signaling pathways [19, 20] and resulting in reduced oxidative stress [32]. In addition, inhibiting FAK activation is also beneficial to alleviate the inflammatory damage [33]. However, the effects and underlying mechanisms of Y15 on the venous oxidative damage induced by CVC indwelling have not been clarified yet. In this study, we investigated the inhibitory effects of Y15 on the oxidative damage of vascular endothelium induced by CVC as well as the underlying mechanisms through in vivo and in vitro models.

The occurrence and development of CVC-related complications are related to oxidative stress and vascular endothelial damage caused by CVC. Gan et al. found that CVC caused a significant increase in serum ROS and MDA levels in a rat model [34]. Other study has also shown that oxidative stress markers were predictors of CVC-related high-risk thrombosis [35]. In addition, infections associated with CVC are usually associated with oxidative stress and inflammation [10, 36]. In this study, CVC was inserted into the junction of the superior vena cava and the right atrium through the right external jugular vein to establish a rabbit model of vein injury caused by CVC insertion and indwelling. The levels of ROS and MDA increased, whereas SOD and NO levels decreased in rabbit serum after CVC was placed. Oxidative stress can damage vascular endothelial cells [37] and trigger an inflammatory response, and excessive ROS is directly involved in the regulation of thrombosis [38]. In this regard, this research also indicates that oxidative stress caused by CVC indwelling is one of the important factors of vascular endothelial cell dysfunction, endothelial cell shedding, massive inflammatory cell infiltration, platelet activation [38, 39], granulation tissue organization after thrombosis, and even scarring organization formation. Thus, the regulation of the levels of oxidative stress markers to reduce the oxidative damage caused by CVC in rabbit serum may be a target for the prevention and treatment of CVC-related complications.

Regulating the redox state of cells via downregulating the levels of oxidative system markers and upregulating the levels of antioxidant system markers is a key measure for preventing vascular endothelial damage, inflammation, and thrombosis induced by oxidative stress. Under normal physiological conditions, oxidative stress markers have the effect of maintaining endothelial function and the balance of oxidation and antioxidation. However, in the ROS-inflammatory cycle, excessive ROS directly oxidizes NO or inhibits the expression of NO synthase by phosphorylating Akt [40], which inhibits the production of NO and causes endothelial dysfunction, thereby triggering the inflammatory process [41, 42]. Upregulation of the levels of NO and endogenous antioxidant enzymes like SOD, catalase, and glutathione peroxidase can lead to ROS detoxification [43] and interrupt the ROS-inflammatory cycle, thus relieving oxidative damage and inflammation. In the present study, our results showed that Y15 increased NO and SOD levels, declined the toxicity of ROS and MDA, and enhanced cell activity. This suggests that Y15 may reduce the oxidative damage and decrease inflammatory cell infiltration induced by CVC, effectively preventing venous thrombosis.

**Figure 7:**
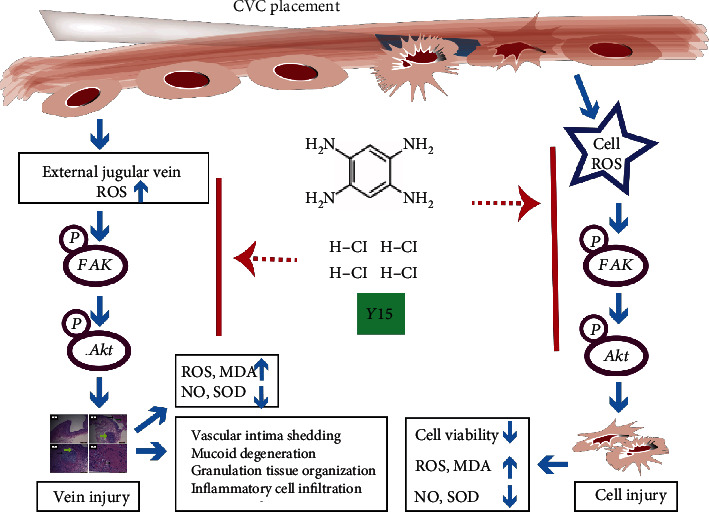
Schematic figure of Y15 inhibiting oxidative damage via the FAK-Akt signaling pathway. In oxidative damage models established by coculturing CVC segments with scratched EA.hy926 cells or inserting and retaining CVC in the vein of rabbits, overproduction of ROS and activation of FAK-Akt signaling triggered the changes in levels of biomarkers of oxidative stress (NO, MDA, and SOD), which resulted in cell injury or vein injury. Y15 alleviated the oxidative damage through inhibition of FAK-Akt pathway activation.

Previous studies have shown that the FAK inhibitor Y15 has antioxidant and anti-inflammatory properties. In the research, to reveal the mechanism of Y15, we investigated the effect of Y15 on FAK and Akt phosphorylation. The imbalance between oxidation and anti-oxidation is one of the important factors in the occurrence and development of oxidative damage [44, 45]. Oxidative stress markers ROS and NO are essential regulators of endothelial function [46]. An excessive amount of ROS would be released after stimulation of vessels, resulting in a decrease in NO levels and endothelial dysfunction [47]. Consistently, we found that after CVC was inserted and indwelled in the vein, ROS, and MDA levels increased, while NO and SOD levels decreased (Figures 2, 5, and 7). It has been shown that overproduction of ROS activates FAK and its downstream signaling molecule Akt [48]. The upregulated phosphorylation level of FAK at Tyr397 further increases the levels of oxidative system markers, such as ROS and MDA, promotes the overexpression of adhesion markers like intercellular adhesion molecule-1 (ICAM-1) and vascular cell adhesion molecule-1 (VCAM-1), and enhances the migration, adhesion, and invasion of inflammatory cells to the damaged vascular endothelium [49]. Moreover, ROS is an important regulator of platelet activation and thrombosis [38, 50]. Overproduction of ROS leads to oxidative damage aggravation [51]. In this study, we observed that there were inflammatory cell infiltration and granulation tissue organization in the external jugular vein at week 4 in the CVC group (Figures 1 and 7). Furthermore, FAK inhibition can reduce the amount of ROS and MDA induced by injury [52]. Inhibiting FAK can also help reduce the level of damage in inflammatory diseases such as rheumatoid arthritis, diabetes, cancer, and neurodegenerative diseases [52, 53]. In addition, Farzaei et al. reported that curcumin reduced oxidative stress damage by inhibiting PI3K/Akt signaling pathway, increased the activity of liver antioxidant enzymes, and reduced liver cell apoptosis [21]. Similarly, An et al. found that the activation of the PI3K/Akt pathway led to oxidative stress, autophagy, and apoptosis in human lung epithelial A549 cells exposed to oxidized black carbon; however, Akt inhibitors significantly inhibited the occurrence of autophagy and apoptosis [48]. Consistently, our observations demonstrate that Y15 inhibited ROS-dependent FAK phosphorylation, reduced the release of ROS and the level of MDA, and increased the level of NO, thereby improving the function of vascular endothelial cells and increasing cell viability (Figures 2–6). In addition, in our study, inhibition of FAK phosphorylation resulted in inhibition of the activation of its downstream signaling molecule Akt, which increased the level of endogenous antioxidant enzyme SOD, and further reduced the degree of cell and vein oxidative damage induced by CVC. Meanwhile, it is reported that FAK activation inhibition plays an important role in anti-inflammation and preventing thrombosis by reducing the overexpression of adhesion molecules ICAM-1 and VCAM-1 and in inhibiting the migration, adhesion, and invasion of inflammatory cells to damaged vascular endothelial cells [54]. Here, our findings also revealed that Y15 significantly reduced inflammatory cell infiltration, venous thrombosis, and granulation tissue organization (Figure 1). Thus, our study confirms that Y15 may have antioxidant and anti-inflammatory effects on vascular oxidative damage caused by CVC and may be preventing CVC-related phlebitis and thrombosis.

## 5. Conclusion

Here, we focused on the effects of FAK inhibitor Y15 on vascular injury induced by CVC and found that Y15 alleviated CVC-induced oxidative damage to blood vessels and prevented blood vessels from CVC-related phlebitis and thrombosis by suppressing FAK-Akt pathway activation. The FAK-Akt pathway may be a promising therapeutic target for the treatment of CVC-related complications.

## Data Availability

The data used to support the findings of this study are available from the corresponding author upon request.
